# *cis*-Cyclo­hexane-1,4-dicarboxylic acid

**DOI:** 10.1107/S1600536809016110

**Published:** 2009-05-14

**Authors:** Yan-Qin Wang, Jia-Bao Weng

**Affiliations:** a Fujian Provincial Key Laboratory for Polymer Materials, College of Chemistry and Materials Science, Fujian Normal University, Fuzhou, Fujian 350007, People’s Republic of China

## Abstract

In the title compound, C_8_H_12_O_4_, the two carboxyl groups are on the same side of the cyclohexane ring and the ring adopts a chair conformation. Adjacent mol­ecules related by an inversion centre are linked by pairs of O—H⋯O hydrogen bonds, forming a zigzag chain along [1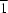 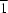].

## Related literature

For related structures, see: Bi *et al.* (2003 ▶, 2004 ▶); Chen *et al.* (2006 ▶); Du *et al.* (2006 ▶); Dunitz & Strickler (1966 ▶); Kurmoo *et al.* (2003 ▶, 2006 ▶); Luger *et al.* (1972 ▶).
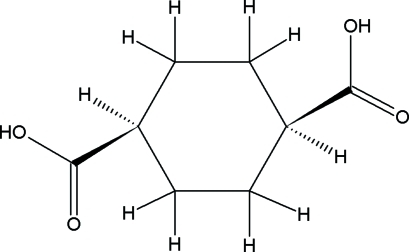


## Experimental

### 

#### Crystal data

C_8_H_12_O_4_
*M* *_r_* = 172.18
Triclinic, 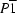

*a* = 5.2912 (6) Å
*b* = 6.2611 (6) Å
*c* = 13.1851 (18) Å
α = 82.505 (10)°
β = 80.309 (11)°
γ = 81.875 (10)°
*V* = 423.70 (9) Å^3^
*Z* = 2
Mo *K*α radiation
μ = 0.11 mm^−1^
*T* = 296 K
0.24 × 0.20 × 0.10 mm

#### Data collection

Bruker SMART CCD area-detector diffractometer
Absorption correction: multi-scan (**SADABS**; Sheldrick, 1996 ▶) *T*_min_ = 0.979, *T*_max_ = 0.989
9807 measured reflections
1925 independent reflections
1222 reflections with *I* > 2σ(*I*)
*R*_int_ = 0.038

#### Refinement

*R*[*F* ^2^ > 2σ(*F* ^2^)] = 0.055
*wR*(*F* ^2^) = 0.129
*S* = 1.05
1925 reflections
118 parameters
H atoms treated by a mixture of independent and constrained refinement
Δρ_max_ = 0.27 e Å^−3^
Δρ_min_ = −0.19 e Å^−3^

### 

Data collection: *SMART* (Bruker, 2007 ▶); cell refinement: *SAINT-Plus* (Bruker, 2007 ▶); data reduction: *SAINT-Plus*; program(s) used to solve structure: *SHELXS97* (Sheldrick, 2008 ▶); program(s) used to refine structure: *SHELXL97* (Sheldrick, 2008 ▶); molecular graphics: *SHELXTL* (Sheldrick, 2008 ▶); software used to prepare material for publication: *SHELXTL*.

## Supplementary Material

Crystal structure: contains datablocks global, I. DOI: 10.1107/S1600536809016110/is2399sup1.cif

Structure factors: contains datablocks I. DOI: 10.1107/S1600536809016110/is2399Isup2.hkl

Additional supplementary materials:  crystallographic information; 3D view; checkCIF report

## Figures and Tables

**Table 1:** Hydrogen-bond geometry (Å, °)

*D*—H⋯*A*	*D*—H	H⋯*A*	*D*⋯*A*	*D*—H⋯*A*
O2—H1⋯O1^i^	0.88 (4)	1.81 (4)	2.684 (2)	178 (4)
O3—H8⋯O4^ii^	1.01 (4)	1.65 (4)	2.658 (2)	175 (4)

Symmetry codes: (i) 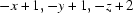; (ii) 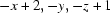.

**Fig. 1.**
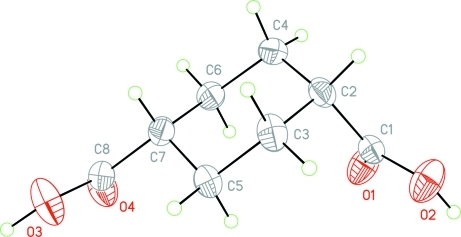
Molecular structure showing 50% probability displacement ellipsoids.

**Fig. 2.**
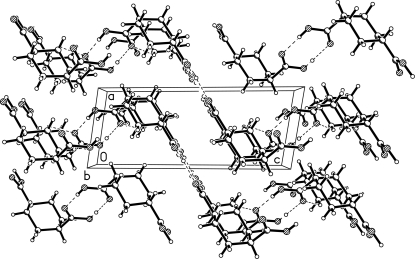
Packing diagram viewed down the b axis, The H-bonds are shown as dotted lines.

## supplementary crystallographic information

### Comment

According to the literatures, there are a few structures incorporating 1,4-*cis*-Cyclohexane dicarboxylic acid (Bi *et al.*, 2004). Although the structures of its isomer in *trans* conformation have been described for more than 40 years (Dunitz & Strickler, 1996; Luger *et al.*, 1972), the structure of the title compound, (I), has only been reported as a co-crystal by Du *et al.* (2006).

According to the results of single X-ray diffraction analysis, there is one complete molecule in the asymmetric unit, and the molecule is in a general position (Fig. 1). The geometry of the molecule is similar to the one observed by Du *et al.* (2006). The bond lengths are comparable to those in its isomers in *trans* conformations (Bi *et al.*, 2003; Chen *et al.*, 2006; Kurmoo *et al.*, 2003, 2006).

Strong hydrogen bonds between two adjacent carboxylate groups link molecules into a zigzag chain along the [111] direction. The zigzag chains are packed into three-dimensional motif (Fig. 2).

### Experimental

C_8_H_12_O_4_ (1 mmol, 172 mg; mixture of *trans*- and *cis*-ACROS) was disloved into 50 ml of CD_3_OD. The solution was stirring and refluxing for 12 h, and the clear solution was allowed to evaporate slowly in the inert atmosphere. Nice plate crystals of the title compound were obtained after 5 days. The crystals were filtered, washed by cool EtOH and dried in the air.

### Refinement

H atoms on O atoms were located in a difference Fourier map and were refined freely. Other H atoms were refined as riding, with C—H = 0.97 or 0.98 Å, and with *U*_iso_(H) = 1.2*U*_eq_(C).

### Crystal data

**Table:**

C_8_H_12_O_4_	*Z* = 2
*M**_r_* = 172.18	*F*(000) = 184
Triclinic, *P*1	*D*_x_ = 1.350 Mg m^−^^3^
Hall symbol: -P 1	Mo *K*α radiation, λ = 0.71073 Å
*a* = 5.2912 (6) Å	Cell parameters from 3560 reflections
*b* = 6.2611 (6) Å	θ = 2.6–26.9°
*c* = 13.1851 (18) Å	µ = 0.11 mm^−^^1^
α = 82.505 (10)°	*T* = 296 K
β = 80.309 (11)°	Plate, colorless
γ = 81.875 (10)°	0.24 × 0.20 × 0.10 mm
*V* = 423.70 (9) Å^3^	

### Data collection

**Table:**

Bruker SMART CCD area-detector diffractometer	1925 independent reflections
Radiation source: fine-focus sealed tube	1222 reflections with *I* > 2σ(*I*)
graphite	*R*_int_ = 0.038
φ and ω scans	θ_max_ = 27.5°, θ_min_ = 3.5°
Absorption correction: multi-scan (*SADABS*; Sheldrick, 1996)	*h* = −6→6
*T*_min_ = 0.979, *T*_max_ = 0.989	*k* = −7→8
9807 measured reflections	*l* = −17→17

### Refinement

**Table:**

Refinement on *F*^2^	Secondary atom site location: difference Fourier map
Least-squares matrix: full	Hydrogen site location: inferred from neighbouring sites
*R*[*F*^2^ > 2σ(*F*^2^)] = 0.055	H atoms treated by a mixture of independent and constrained refinement
*wR*(*F*^2^) = 0.129	*w* = 1/[σ^2^(*F*_o_^2^) + (0.038*P*)^2^ + 0.2496*P*] where *P* = (*F*_o_^2^ + 2*F*_c_^2^)/3
*S* = 1.05	(Δ/σ)_max_ < 0.001
1925 reflections	Δρ_max_ = 0.27 e Å^−^^3^
118 parameters	Δρ_min_ = −0.19 e Å^−^^3^
0 restraints	Extinction correction: *SHELXL97* (Sheldrick, 2008), Fc^*^=kFc[1+0.001xFc^2^λ^3^/sin(2θ)]^-1/4^
Primary atom site location: structure-invariant direct methods	Extinction coefficient: 0.057 (12)

### Special details

**Table:**

Geometry. All e.s.d.'s (except the e.s.d. in the dihedral angle between two l.s. planes) are estimated using the full covariance matrix. The cell e.s.d.'s are taken into account individually in the estimation of e.s.d.'s in distances, angles and torsion angles; correlations between e.s.d.'s in cell parameters are only used when they are defined by crystal symmetry. An approximate (isotropic) treatment of cell e.s.d.'s is used for estimating e.s.d.'s involving l.s. planes.
Refinement. Refinement of *F*^2^ against ALL reflections. The weighted *R*-factor *wR* and goodness of fit *S* are based on *F*^2^, conventional *R*-factors *R* are based on *F*, with *F* set to zero for negative *F*^2^. The threshold expression of *F*^2^ > σ(*F*^2^) is used only for calculating *R*-factors(gt) *etc*. and is not relevant to the choice of reflections for refinement. *R*-factors based on *F*^2^ are statistically about twice as large as those based on *F*, and *R*- factors based on ALL data will be even larger.

### Fractional atomic coordinates and isotropic or equivalent isotropic displacement parameters (Å^2^)

**Table:**

	*x*	*y*	*z*	*U*_iso_*/*U*_eq_	
O1	0.4359 (3)	0.5154 (3)	0.88398 (12)	0.0583 (5)	
O2	0.2703 (4)	0.2964 (3)	1.01503 (13)	0.0620 (5)	
O3	0.7370 (4)	−0.1284 (3)	0.57741 (15)	0.0709 (6)	
O4	0.8260 (3)	0.2120 (3)	0.55122 (14)	0.0632 (5)	
C1	0.2929 (4)	0.3798 (3)	0.91784 (16)	0.0386 (5)	
C2	0.1278 (4)	0.2898 (4)	0.85544 (16)	0.0420 (5)	
H2	−0.0488	0.3021	0.8932	0.050*	
C3	0.2176 (4)	0.0476 (3)	0.84796 (17)	0.0457 (6)	
H3A	0.2410	−0.0240	0.9161	0.055*	
H3B	0.0853	−0.0174	0.8242	0.055*	
C4	0.1185 (4)	0.4153 (4)	0.74843 (17)	0.0493 (6)	
H4A	−0.0264	0.3782	0.7208	0.059*	
H4B	0.0878	0.5694	0.7557	0.059*	
C5	0.4695 (4)	0.0121 (3)	0.77409 (16)	0.0411 (5)	
H5A	0.5175	−0.1420	0.7694	0.049*	
H5B	0.6056	0.0664	0.8006	0.049*	
C6	0.3638 (4)	0.3713 (4)	0.67093 (17)	0.0457 (6)	
H6A	0.3350	0.4396	0.6028	0.055*	
H6B	0.5029	0.4346	0.6907	0.055*	
C7	0.4420 (4)	0.1286 (3)	0.66664 (16)	0.0411 (5)	
H7	0.3028	0.0710	0.6423	0.049*	
C8	0.6862 (4)	0.0769 (4)	0.59270 (16)	0.0432 (5)	
H1	0.369 (7)	0.355 (6)	1.048 (3)	0.106 (12)*	
H8	0.898 (8)	−0.163 (6)	0.526 (3)	0.128 (13)*	

### Atomic displacement parameters (Å^2^)

**Table:**

	*U*^11^	*U*^22^	*U*^33^	*U*^12^	*U*^13^	*U*^23^
O1	0.0785 (12)	0.0610 (11)	0.0431 (9)	−0.0335 (10)	−0.0145 (8)	−0.0002 (8)
O2	0.0795 (13)	0.0717 (12)	0.0413 (10)	−0.0360 (10)	−0.0111 (9)	0.0020 (8)
O3	0.0759 (13)	0.0525 (11)	0.0741 (13)	−0.0054 (9)	0.0251 (10)	−0.0191 (9)
O4	0.0571 (11)	0.0595 (11)	0.0685 (12)	−0.0124 (9)	0.0167 (9)	−0.0190 (9)
C1	0.0371 (11)	0.0377 (11)	0.0396 (12)	−0.0004 (9)	−0.0016 (9)	−0.0091 (9)
C2	0.0299 (10)	0.0512 (13)	0.0452 (12)	−0.0048 (9)	0.0017 (9)	−0.0163 (10)
C3	0.0454 (12)	0.0447 (13)	0.0476 (13)	−0.0145 (10)	0.0045 (10)	−0.0131 (10)
C4	0.0422 (12)	0.0544 (14)	0.0531 (14)	0.0066 (10)	−0.0150 (10)	−0.0168 (11)
C5	0.0437 (12)	0.0358 (11)	0.0422 (12)	−0.0034 (9)	0.0003 (9)	−0.0084 (9)
C6	0.0490 (13)	0.0490 (13)	0.0378 (12)	0.0017 (10)	−0.0098 (10)	−0.0038 (10)
C7	0.0377 (11)	0.0491 (13)	0.0383 (11)	−0.0058 (9)	−0.0051 (9)	−0.0122 (9)
C8	0.0450 (12)	0.0483 (14)	0.0366 (11)	−0.0024 (10)	−0.0057 (9)	−0.0097 (10)

### Geometric parameters (Å, °)

**Table:**

O1—C1	1.209 (2)	C3—H3B	0.9700
O2—C1	1.312 (3)	C4—C6	1.527 (3)
O2—H1	0.88 (4)	C4—H4A	0.9700
O3—C8	1.310 (3)	C4—H4B	0.9700
O3—H8	1.01 (4)	C5—C7	1.526 (3)
O4—C8	1.217 (3)	C5—H5A	0.9700
C1—C2	1.503 (3)	C5—H5B	0.9700
C2—C4	1.528 (3)	C6—C7	1.523 (3)
C2—C3	1.534 (3)	C6—H6A	0.9700
C2—H2	0.9800	C6—H6B	0.9700
C3—C5	1.520 (3)	C7—C8	1.505 (3)
C3—H3A	0.9700	C7—H7	0.9800
			
C1—O2—H1	110 (2)	H4A—C4—H4B	107.6
C8—O3—H8	114 (2)	C3—C5—C7	110.71 (18)
O1—C1—O2	121.6 (2)	C3—C5—H5A	109.5
O1—C1—C2	124.7 (2)	C7—C5—H5A	109.5
O2—C1—C2	113.66 (19)	C3—C5—H5B	109.5
C1—C2—C4	113.11 (18)	C7—C5—H5B	109.5
C1—C2—C3	109.99 (18)	H5A—C5—H5B	108.1
C4—C2—C3	111.41 (17)	C7—C6—C4	111.18 (19)
C1—C2—H2	107.3	C7—C6—H6A	109.4
C4—C2—H2	107.3	C4—C6—H6A	109.4
C3—C2—H2	107.3	C7—C6—H6B	109.4
C5—C3—C2	111.67 (17)	C4—C6—H6B	109.4
C5—C3—H3A	109.3	H6A—C6—H6B	108.0
C2—C3—H3A	109.3	C8—C7—C6	113.13 (18)
C5—C3—H3B	109.3	C8—C7—C5	109.85 (18)
C2—C3—H3B	109.3	C6—C7—C5	111.08 (17)
H3A—C3—H3B	107.9	C8—C7—H7	107.5
C6—C4—C2	114.13 (18)	C6—C7—H7	107.5
C6—C4—H4A	108.7	C5—C7—H7	107.5
C2—C4—H4A	108.7	O4—C8—O3	122.7 (2)
C6—C4—H4B	108.7	O4—C8—C7	123.5 (2)
C2—C4—H4B	108.7	O3—C8—C7	113.8 (2)
			
O1—C1—C2—C4	−10.6 (3)	C2—C4—C6—C7	51.0 (2)
O2—C1—C2—C4	169.74 (18)	C4—C6—C7—C8	−178.86 (18)
O1—C1—C2—C3	114.7 (2)	C4—C6—C7—C5	−54.8 (2)
O2—C1—C2—C3	−65.0 (2)	C3—C5—C7—C8	−175.50 (17)
C1—C2—C3—C5	−74.0 (2)	C3—C5—C7—C6	58.6 (2)
C4—C2—C3—C5	52.3 (2)	C6—C7—C8—O4	9.4 (3)
C1—C2—C4—C6	74.9 (2)	C5—C7—C8—O4	−115.3 (2)
C3—C2—C4—C6	−49.6 (2)	C6—C7—C8—O3	−171.16 (19)
C2—C3—C5—C7	−57.3 (2)	C5—C7—C8—O3	64.1 (2)

### Hydrogen-bond geometry (Å, °)

**Table:**

*D*—H···*A*	*D*—H	H···*A*	*D*···*A*	*D*—H···*A*
O2—H1···O1^i^	0.88 (4)	1.81 (4)	2.684 (2)	178 (4)
O3—H8···O4^ii^	1.01 (4)	1.65 (4)	2.658 (2)	175 (4)

Symmetry codes: (i) −*x*+1, −*y*+1, −*z*+2; (ii) −*x*+2, −*y*, −*z*+1.
